# A multi-module enhanced YOLOv8 framework for accurate AO classification of distal radius fractures: SCFAST-YOLO

**DOI:** 10.3389/fmed.2025.1635016

**Published:** 2025-08-20

**Authors:** Yu Wang, Haifu Sun, Tiankai Jiang, JunFeng Shi, Qin Wang, Hongwei Yang, Yusen Qiao

**Affiliations:** ^1^Department of Orthopaedics, The First Affiliated Hospital of Soochow University, Suzhou, China; ^2^School of medicine, Nantong University, Nantong, Jiangsu, China; ^3^Department of Orthopaedics, Affiliated Nantong Hospital 3 of Nantong University, Nantong, China; ^4^Department of Orthopaedics, Nantong Third People's Hospital, Nantong, China

**Keywords:** distal radius fractures, YOLOv8, C2f-Faster-EMA, TADDH, FDPN

## Abstract

**Introduction:**

CT-based classification of distal ulnar-radius fractures requires precise detection of subtle features for surgical planning, yet existing methods struggle to balance accuracy with clinical efficiency. This study aims to develop a lightweight architecture that achieves accurate AO (Arbeitsgemeinschaft für Osteosynthesefragen) typing[an internationally recognized fracture classification system based on fracture location, degree of joint surface involvement, and comminution, divided into three major categories: A (extra-articular), B (partially intra-articular), and C (completely intra-articular)] while maintaining real-time performance. In this task, the major challenges are capturing complex fracture morphologies without compromising detection speed and ensuring precise identification of small articular fragments critical for surgical decision-making.

**Methods:**

We propose SCFAST-YOLO framework to address these challenges. Its first contribution is introducing the SCConv module, which integrates Spatial and Channel Reconstruction Units to systematically reduce feature redundancy while preserving discriminative information essential for detecting subtle articular fragments. Secondly, we develop the C2f-Faster-EMA module that preserves fine-grained spatial details through optimized information pathways and statistical feature aggregation. Third, our Feature-Driven Pyramid Network facilitates multi-resolution feature fusion across scales for improved detection. Finally, we implement a Target-Aware Dual Detection Head that employs task decomposition to enhance localization precision.

**Results and discussion:**

Evaluated on our FHSU-DRF dataset (332 cases, 1,456 CT sequences), SCFAST-YOLO achieves 91.8% mAP@0.5 and 87.2% classification accuracy for AO types, surpassing baseline YOLOv8 by 2.1 and 2.3 percentage points respectively. The most significant improvements appear in complex Type C fractures (3.2 percentage points higher classification accuracy) with consistent average recall of 0.85–0.88 across all fracture patterns. The model maintains real-time inference (52.3 FPS) while reducing parameters, making it clinically viable. Extensive qualitative and quantitative results demonstrate the advantages of our approach. Additionally, we show the broader clinical applications of SCFAST-YOLO in enhancing consistency and efficiency in trauma care.

## 1 Introduction

With the continuous development of deep learning, more and more models are applied to medical image processing (2–8), including lung nodule detection (9), breast cancer screening (10) and brain tumor segmentation (11). Detection and definition of ulnar-radial fracture is a classical problem in medical imaging and a common stereo object detection challenge in computer vision, and the detection of ulnar-radial fracture using a deep learning model can greatly improve the accuracy and efficiency of the detection and reduce the human error. Recent studies have demonstrated that deep learning frameworks can automatically identify and classify different types of ulnar-radial fractures (12), significantly improving diagnostic efficiency and reducing inter-observer variability.

Detection and typing of distal ulnar radius fractures using deep learning models also faces multiple challenges (13–16, 16). Firstly, the medical images used for ulnar radius fracture typing definition are mainly CT 3D reconstructed images (17), which include 3D fracture images with various angles, and they are significantly different in features and distribution compared with traditional imaging data. The ulnar-radial fracture images are more diverse, including different morphologies of fracture lines, free bone fragments, and variations in metacarpophalangeal joint anatomy. This diversity makes the detection of distal ulnar radius fractures more challenging. Second, the morphology of the fracture site in distal ulnar radius fracture images varies widely. The images may contain simple fractures, wedge fractures, and comminuted fractures of the ulna and radius, and the differences in fracture morphology increase the complexity of the detection task. In addition, the anatomical structures involved in distal ulnar radius images are complex. The morphology of the fractured free bone mass in a complete CT image of a distal ulnar radius fracture may be similar to that of the metacarpal-wrist constituent bones, making it difficult to differentiate between them, thus further complicating the detection task. In addition, CT images reconstructed in three dimensions may result in loss of detail and incomplete information, thus hindering accurate fracture detection and typing definition. Finally, the quality of ulnar radius fracture images can be affected by the patient's posture, the degree of injury sustained, and the adjustment of CT image processing parameters. For example, different parameters of CT image window width can affect image quality and consistency. Together, these factors contribute to the difficulty of image detection, diagnosis and typing definition of distal ulnar radius fractures (18, 19).

Recent advances in deep learning (20), particularly with the YOLO (You Only Look Once) (21, 22) family of models, have demonstrated promising results for medical imaging applications. The latest iteration, YOLOv8 (20, 23), offers improvements in both accuracy and efficiency compared to its predecessors. However, its application to the specialized domain of fracture classification requires targeted architectural modifications to address the unique challenges presented by orthopedic imaging. Existing approaches still struggle with optimal feature extraction for complex fracture patterns, computational inefficiency due to redundancy in neural network architectures, and difficulties in effectively handling the variability in fracture fragment sizes encountered in clinical practice.

In order to address these challenges and improve the detection and typing definition of distal ulnar radius fractures, this paper proposes the SCFAST-YOLO modeling framework. Our approach introduces four complementary innovations to the YOLOv8 framework. The SCConv module integrates Spatial Reconstruction Unit (SRU) (24) and Channel Reconstruction Unit (CRU) to systematically reduce redundancy while preserving discriminative features critical for detecting subtle articular fragments. The C2f-Faster-EMA module preserves fine-grained spatial information through optimized information pathways and statistical feature aggregation. The Feature-Driven Pyramid Network facilitates multi-resolution feature fusion across scales, while the Target-Aware Dual Detection Head (25) employs task decomposition to enhance localization precision for complex fracture features.

The main contributions of this paper are as follows:

1. An innovative SCFAST-YOLO framework for fracture typing detection of distal ulnar radius fractures is proposed in this study, achieving state-of-the-art performance while maintaining real-time inference capabilities.
2. The feature extraction capability of the model is enhanced by the introduction of the SCConv module, which effectively reduces redundancy in both spatial and channel dimensions while preserving essential features. The introduction of the C2f-Faster-EMA module enables the model to better maintain multi-scale contextual information, capturing the hierarchical structure of complex fracture patterns. The addition of the FDPN module facilitates sophisticated feature fusion mechanisms that integrate information across scales, while the TADDH module improves detection performance through task-specific decomposition.
3. Experiments on our FHSU-DRF dataset (332 cases, 1,456 CT sequences) show that this approach significantly improves the recognition ability of the YOLO model in fracture detection and typing definition. SCFAST-YOLO achieves 91.8% mAP@0.5 and 87.2% classification accuracy for AO types, surpassing baseline YOLOv8 by 2.1 and 2.3 percentage points respectively, with most significant enhancements observed in complex Type C fractures (3.2 percentage points improvement in classification accuracy).

## 2 Related work

### 2.1 YOLO

Neural convolutional networks have achieved important milestones in the development of deep learning, and YOLO is one of the very important models. Due to its simple design, efficient network and excellent detection capability, the YOLO model is widely used in the field of object detection. Liu et al. demonstrated the application of YOLOv5 for industrial defect detection, enabling efficient defect identification through real-time monitoring of production lines (26). Jha et al. showed that YOLOv4 performs well in the detection of foreign objects in food and can effectively reduce the cost and time of manual inspection (27). Aldakheel EA implemented real-time detection of plant diseases using the YOLOv4 model, and the experimental results show that the method outperforms traditional image processing techniques in terms of accuracy and speed (28). In most approaches, the effectiveness of the YOLO framework is usually enhanced by adding attention mechanisms and other techniques. However, in deep learning, these methods often run into the problem of vanishing gradients as the depth of the model increases.

### 2.2 Deep learning methods in medical image processing

In recent years, with significant advances in deep learning, more and more deep learning models have been applied to medical image processing. Zhang et al. proposed a YOLO-based liver tumor detection method, which significantly improved the detection accuracy by optimizing the model parameters (29). In lung CT imaging, Ren Y et al. utilized a YOLO framework-based model for the automatic detection of lung nodules (30). By training the model to recognize nodule features, the study demonstrated superior accuracy and processing speed compared to traditional methods. Santos C's team applied YOLO to retinal image analysis, achieving high-accuracy identification of early-stage diabetic retinopathy (31). YOLO also exhibits outstanding performance in detecting both superficial and deep-seated organs. For instance, Nurmaini S et al. employed an improved YOLO model to automate the assessment of fetal cardiac enlargement; the model demonstrated robust detection capabilities, providing a reliable computer-assisted tool for ultrasound physicians during prenatal screening (32). In a recent study, YOLOv5 was used to automate the detection of skin lesions, and the results showed that the model excelled in the identification of different types of skin cancers and was effective in assisting doctors in making a diagnosis (33).

For the task of distal ulnar radius fracture typing detection, the **SCConv** and **C2f-Faster-EMA** modules are introduced in this paper to address the problem of indistinguishable fracture morphology and imaging differences between different angles at the distal ulnar radius fracture site. These modules effectively improve the ability of the model to extract features and enhance the performance of the model in distal ulnar radius typing detection.

### 2.3 Typing of distal ulnar radius fractures

Distal ulnar radius fracture typing detection is a valuable task in the field of computer vision and medical imaging. Its main challenges are the complex geometry of distal ulnar radius fractures and the small size of currently available datasets. At present, there are no comparable large, high-quality datasets for distal ulnar–radius fractures. Existing datasets often have an insufficient number of available records and small sample sizes, as data sharing is severely restricted by patient information (34). The comparability and reliability of study results are affected by significant differences in acquisition standards, image quality, and labeling methods between different datasets (35). Despite the large number of studies based on publicly accessible datasets, the lack of sufficient empirical clinical evidence calls into question the reliability and validity of the models in actual clinical use (36). Despite the significant clinical value of automated fracture classification systems, the field continues to face numerous challenges, including geometric complexity, data scarcity, and insufficient clinical validation. These limitations underscore the pressing need for methodological innovation to surmount dataset constraints while ensuring clinical applicability. In this context, further exploration of deep learning techniques, such as data augmentation, domain adaptation, and model design informed by clinical information, will drive the development of reliable fracture classification systems with practical applications.

## 3 Materials and methods

This section presents a comprehensive examination of our enhanced YOLOv8-based distal radius fracture detection architecture and provides a detailed analysis of the underlying operational mechanisms. Distal radius fractures were classified into three categories according to AO classification (1). We systematically investigate the structural components that form the foundation of our approach, introducing four pivotal modules that constitute our improvements: the SCConv module, C2f-Faster-EMA module, TADDH detection head, and FDPN feature fusion network.

### 3.1 AO classification

**Figure 1:**
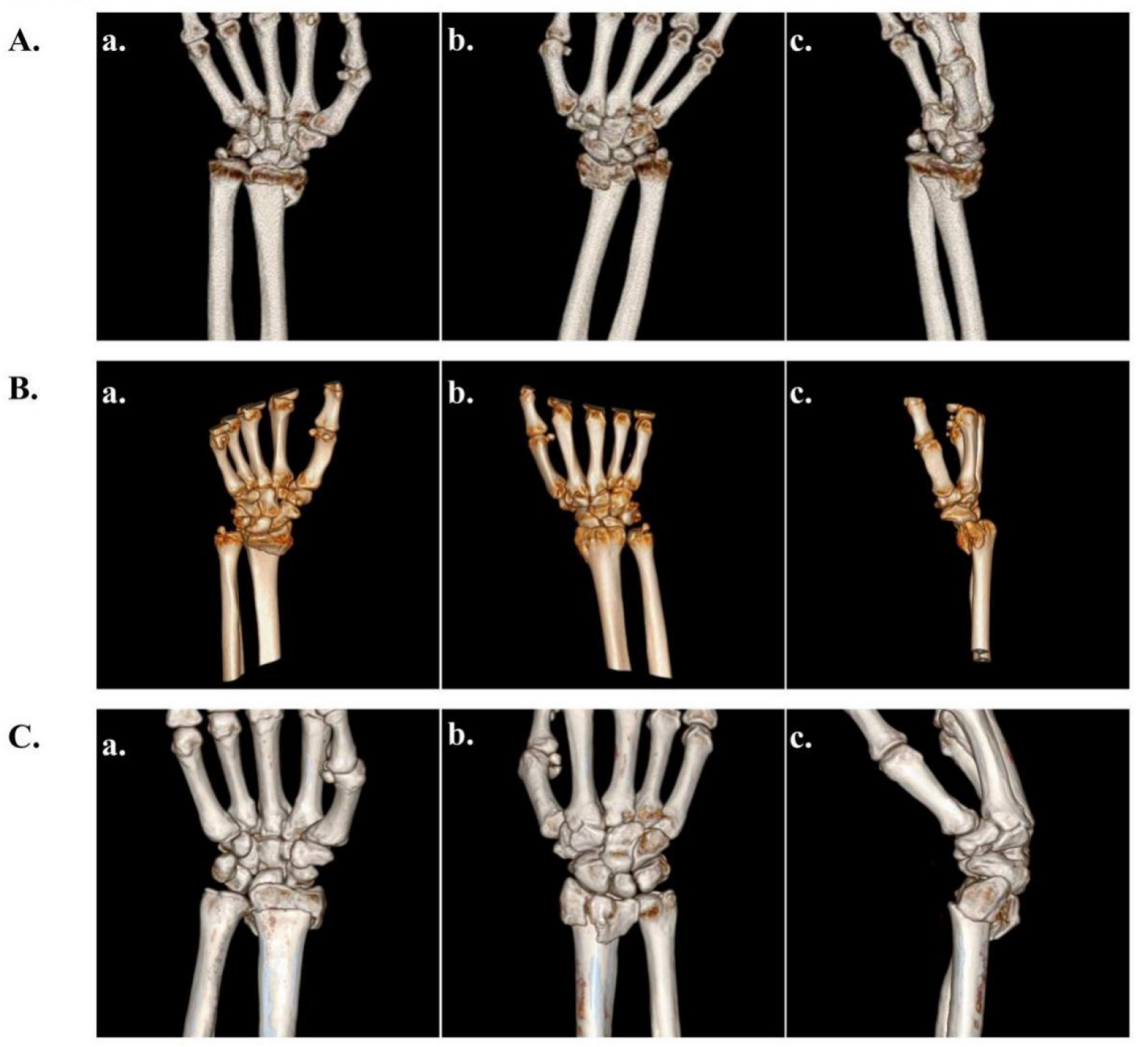
AO typing of distal radius fractures. **(A)** Frontal and lateral 3D reconstruction of CT images of extra-articular fractures (Type A). **(B)** Frontal and lateral 3D reconstruction of CT images of partial intra-articular fractures (Type B). **(C)** Frontal and lateral 3D reconstruction of CT images of complete intra-articular fractures (Type C).

In this article, we have analyzed and refer to the existing distal radius fracture typing guidelines and clinical practice recommendations to adopt AO typing to classify the injured radius with CT images (37). The AO typing will be utilized for the diagnosis and identification of the injured radius, which will be classified into three distinct types: Type A (extra-articular fracture), Type B (partial extra-articular fracture), and Type C (complete extra-articular fracture) (38). This classification will serve as a foundation for the subsequent treatment and prognosis of distal radius fractures (Figure 1).

### 3.2 YOLOv8 framework

YOLOv8, released in early 2023, represents a significant advancement in real-time object detection frameworks. The architecture is available in five scale variants—YOLOv8n, YOLOv8s, YOLOv8m, YOLOv8l, and YOLOv8x—each designed to address specific computational constraints and accuracy requirements across diverse computer vision tasks. This scalability enables practitioners to select the most appropriate model configuration that optimizes the trade-off between detection accuracy and computational efficiency for their particular application context.

The YOLOv8n model, being the most lightweight implementation in the series, comprises four fundamental components: the input layer for image preprocessing, the backbone network for feature extraction, the neck for multi-scale feature fusion, and the head for prediction generation. The backbone network incorporates specialized C2f and CBS modules that systematically extract hierarchical features from input images. The C2f module employs a lightweight design that effectively captures fine-grained gradient information, substantially enhancing the model's capability to detect small objects. Complementing this, the CBS module integrates convolutional operations with batch normalization and activation functions, significantly improving feature representation learning.

The neck architecture implements a bidirectional feature integration strategy by combining Feature Pyramid Network (FPN) (8) with Path Aggregation Network (PAN) (39). This sophisticated structure facilitates effective information flow between different resolution scales, enabling the model to simultaneously capture both fine-grained details and broader contextual information. The detection head generates bounding box coordinates, objectness scores, and class probabilities, with Non-Maximum Suppression (NMS) (40) applied during post-processing to eliminate redundant detections.

### 3.3 Enhanced architecture: YOLOv8-SCFAST

Our enhanced architecture, designated as YOLOv8-SCFAST, fundamentally restructures the traditional YOLOv8 framework by systematically replacing conventional Conv modules with our proposed SCConv modules. These SCConv units implement a channel-wise attention mechanism that begins by applying global average pooling to compress spatial information across feature maps, generating compact channel descriptors. These descriptors subsequently undergo transformation through fully connected layers and non-linear activation functions to derive adaptive channel-specific weights, which are then applied to the original feature representations, effectively recalibrating channel importance.

Our architectural optimization extends beyond the backbone network to encompass strategic modifications to both the neck components and the critical C2f module of YOLOv8. These comprehensive refinements yield a significantly more compact model with substantially reduced parameter count and memory footprint, making it particularly suitable for deployment in resource-constrained environments such as mobile healthcare systems or emergency room diagnostics (replacing the previous reference to “vehicle detection systems” to align with the medical application of this study).

A key innovation in our approach is the development of the C2f-Faster-EMA module, which represents a substantial evolution of the original C2f structure. This module replaces the conventional Bottleneck architecture with our proposed FasterBlock (41) component, which achieves enhanced computational efficiency while maintaining feature representation capacity. The FasterBlock implements an optimized information flow pathway that reduces redundant operations while preserving essential feature extraction capabilities.

To address the challenge of diminishing localization precision for small objects that typically occurs with increasing network depth, we incorporate the Exponential Moving Average (EMA) module. This component maintains a continuously updated statistical representation of features across multiple scales, effectively preserving fine-grained spatial information that would otherwise be lost during downsampling operations and feature aggregation processes.

**Figure 2:**
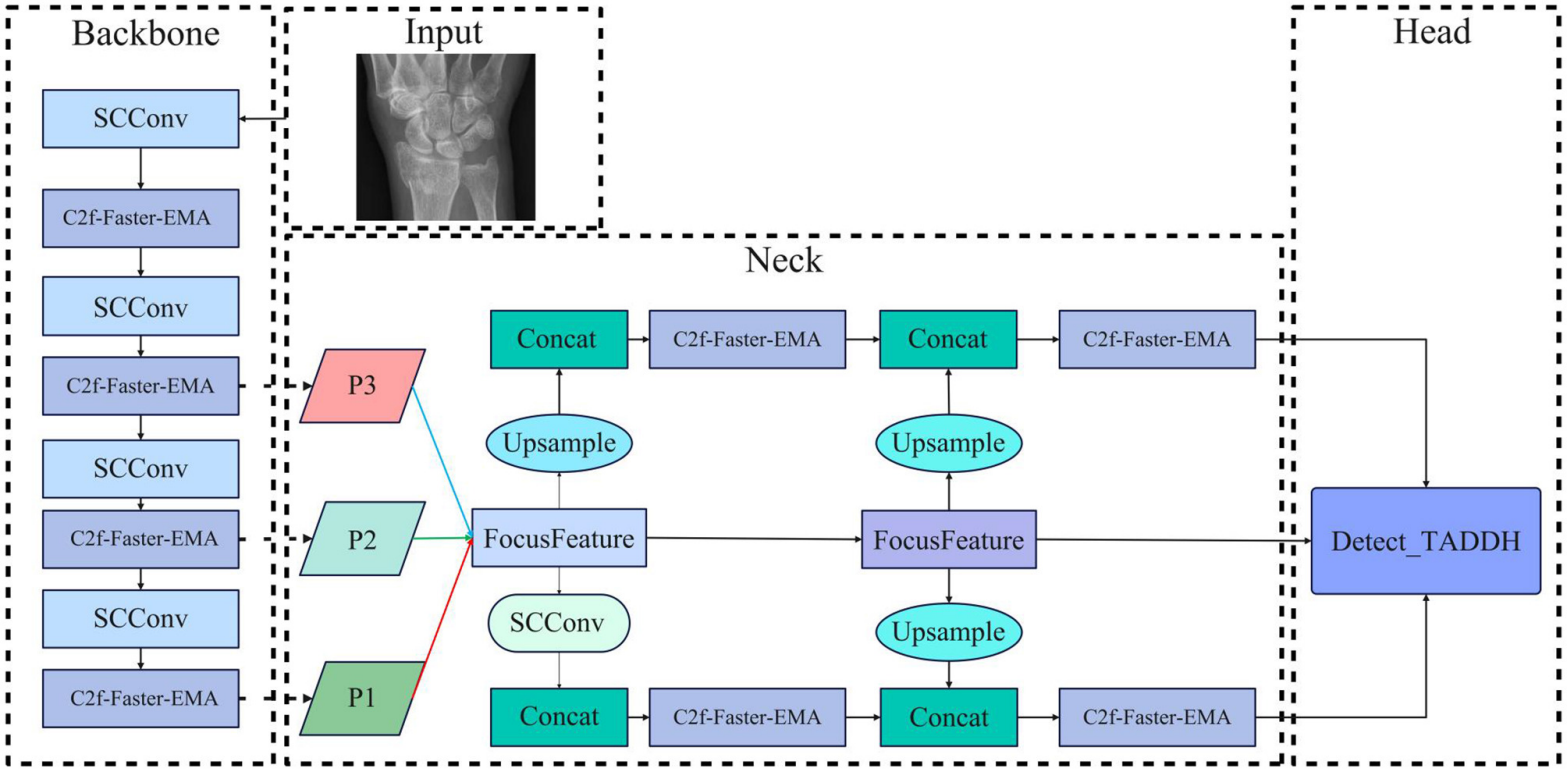
Overview structure of YOLOv8-SCFAST, illustrating the key components and information flow.

Further augmenting the model's detection capabilities, we integrate the Feature-Driven Pyramid Network (FDPN) module alongside the Target-Aware Dual Detection Head (TADDH). This architectural combination enables our model to effectively learn and represent multi-scale features with scale-specific characteristics. The FDPN facilitates adaptive feature fusion across different resolution levels, while TADDH implements parallel detection pathways optimized for targets of varying scales (Figure 2).

### 3.4 SCConv module

Convolutional neural networks (42) have demonstrated remarkable performance in computer vision tasks; however, they typically demand substantial computational resources and memory capacity, which significantly constrains their deployment in resource-limited environments. Extensive research has revealed inherent redundancies in deep networks, manifested both in model parameters and feature representations across spatial and channel dimensions. To address these limitations, we propose the SCConv module—a lightweight yet effective architectural component designed to enhance feature representation while reducing computational overhead.

The SCConv module comprises two complementary components: the Spatial Reconstruction Unit (SRU) and the Channel Reconstruction Unit (CRU), which operate sequentially to refine feature representations. Given an input feature map *X* within a bottleneck residual block, the processing pipeline begins with the SRU, which systematically reconstructs and refines spatial information. The refined spatial features are subsequently processed by the CRU, which performs adaptive channel-wise feature recalibration to emphasize informative channels while suppressing less relevant ones.

#### 3.4.1 Spatial reconstruction unit

The SRU addresses spatial redundancy by leveraging Group Normalization's scaling factor γ to distinguish information-rich features. We generate an information weight map *W*_γ_ normalized to (0, 1) via Sigmoid activation, then apply a threshold (0.5) to separate input feature map *X* into information-rich (*X*_*w*1_) and less informative (*X*_*w*2_) components. Through cross-reconfiguration, these components enhance each other via element-wise addition, producing reconstructed maps $X_{w1}^{\prime }$ and $X_{w2}^{\prime }$ that are concatenated to form the refined output *X*_ω_:


(1) $$\{\begin{array}{l} X_{w1}=W_{1}⊙X,\\ X_{w2}=W_{2}⊙X,\\ {X^{\prime }}_{w1}=X_{w1}⊕X_{w2},\\ {X^{\prime }}_{w2}=X_{w2}⊕X_{w1},\\ X_{w}=\text{Concat}({X^{\prime }}_{w1},{X^{\prime }}_{w2}), \end{array}$$

This design reduces spatial redundancy while preserving essential feature representation.

#### 3.4.2 Channel reconstruction unit

The CRU minimizes channel redundancy through a dual-path architecture. The primary path generates *X*_*up*_ using efficient group-wise (GWC) and point-wise convolutions (PWC), while the secondary path produces *X*_*low*_ via 1 × 1 PWC operations. We implement a simplified Selective Kernel mechanism where global average pooling extracts channel descriptors from both paths, which then undergo soft attention to compute importance vectors β_1_ and β_2_. The final output is formed by weighted combination:


(2) $$\{\begin{array}{l} X_{w}=\text{Concat}(X_{1},X_{2},\ldots ,X_{n}),\\ X_{up}=\text{GWC}(X_{w})+\text{PWC}(X_{w}),\\ X_{low}={\text{PWC}}_{1\times 1}(X_{w}),\\ Y_{1}=\text{GAP}(X_{up}),\\ Y_{2}=\text{GAP}(X_{low}),\\ \beta_{1},\beta_{2}=\text{SoftChannelAttention}(Y_{1},Y_{2}),\\ Y=\beta_{1}\cdot Y_{1}+\beta_{2}\cdot Y_{2}, \end{array}$$

By replacing standard convolutions with our SCConv modules (sequential SRU + CRU), we effectively reduce both spatial and channel redundancies, enhancing feature representation with improved computational efficiency.

### 3.5 C2f-faster-EMA module

**Figure 3:**
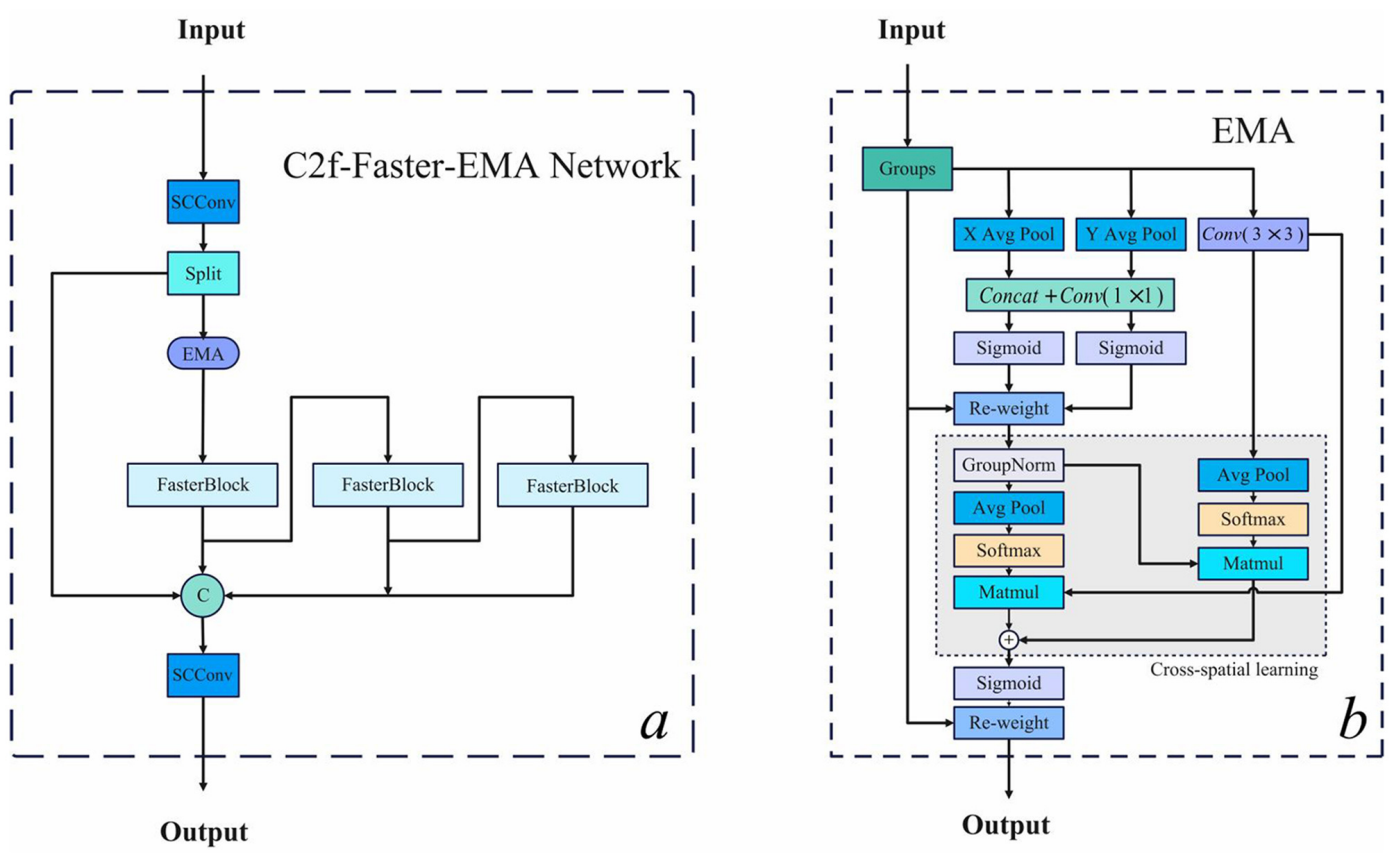
(a) C2f-Faster-EMA module architecture incorporating FasterNet components for optimized information flow; (b) The Efficient Multi-scale Attention (EMA) mechanism with parallel branches for global and local feature extraction.

In the traditional YOLOv8 model, the C3 module has evolved into the C2f module, strategically positioned within the deeper layers of the backbone network. This architectural decision facilitates the fusion of shallow feature maps—characterized by higher resolution but limited semantic information—with deep feature maps that possess lower resolution but enriched semantic content. Our proposed C2f-Faster-EMA model, as illustrated in Figure 3(a), represents a substantial evolution of this architecture by integrating FasterNet components with an Efficient Multi-scale Attention (EMA) (43) mechanism shown in Figure 3(b). FasterNet, introduced at the CVPR 2023 conference, addresses the critical need for reduced latency while preserving computational performance through its architectural framework that integrates two complementary convolutional operations: Pointwise Convolution (PWConv) and Partitioned Convolution (PConv). These manifest in two distinct configurations: T-Convolution and dual convolutional patterns operating in parallel. Each fundamental unit within this architecture comprises a composite structure consisting of one PConv and two PWConv configurations, with normalization and activation layers serving as critical processing elements. T-Convolution distinguishes itself through its centrality-biased weighting scheme, assigning elevated importance to the central position of the convolutional kernel, facilitating more efficient computational processing by concentrating resources on the most informative spatial regions.

Complementing this architecture, the EMA module optimizes the distribution of computational resources across diverse feature combinations through parallel processing pathways that enhance the model's capacity for extracting both global and local features. Specifically, the input feature map is divided along the channel dimension into two branches: the first applies 1D global pooling followed by 1 × 1 convolution to efficiently capture global contextual information, while the second utilizes 3 × 3 convolution to extract localized spatial features. The outputs from these complementary branches are subsequently combined through matrix multiplication to generate an attention map, which integrates with the original input through a residual connection. This strategic integration enables our model to effectively capture multi-scale contextual information while maintaining the computational advantages of the FasterNet framework, addressing the challenge of diminishing localization precision for small objects that typically occurs with increasing network depth by maintaining a continuously updated statistical representation of features across scales. Its structure is shown in the Figure 3.

Ablation results (Section 4.5) confirm that incorporating EMA yields a +0.5% mAP gain and +0.4% classification accuracy improvement compared to using FasterNet alone, quantitatively validating EMAs ability to preserve fine-grained spatial details.

### 3.6 FDPN and TADDH modules

#### 3.6.1 Feature-driven pyramid network

**Figure 4:**
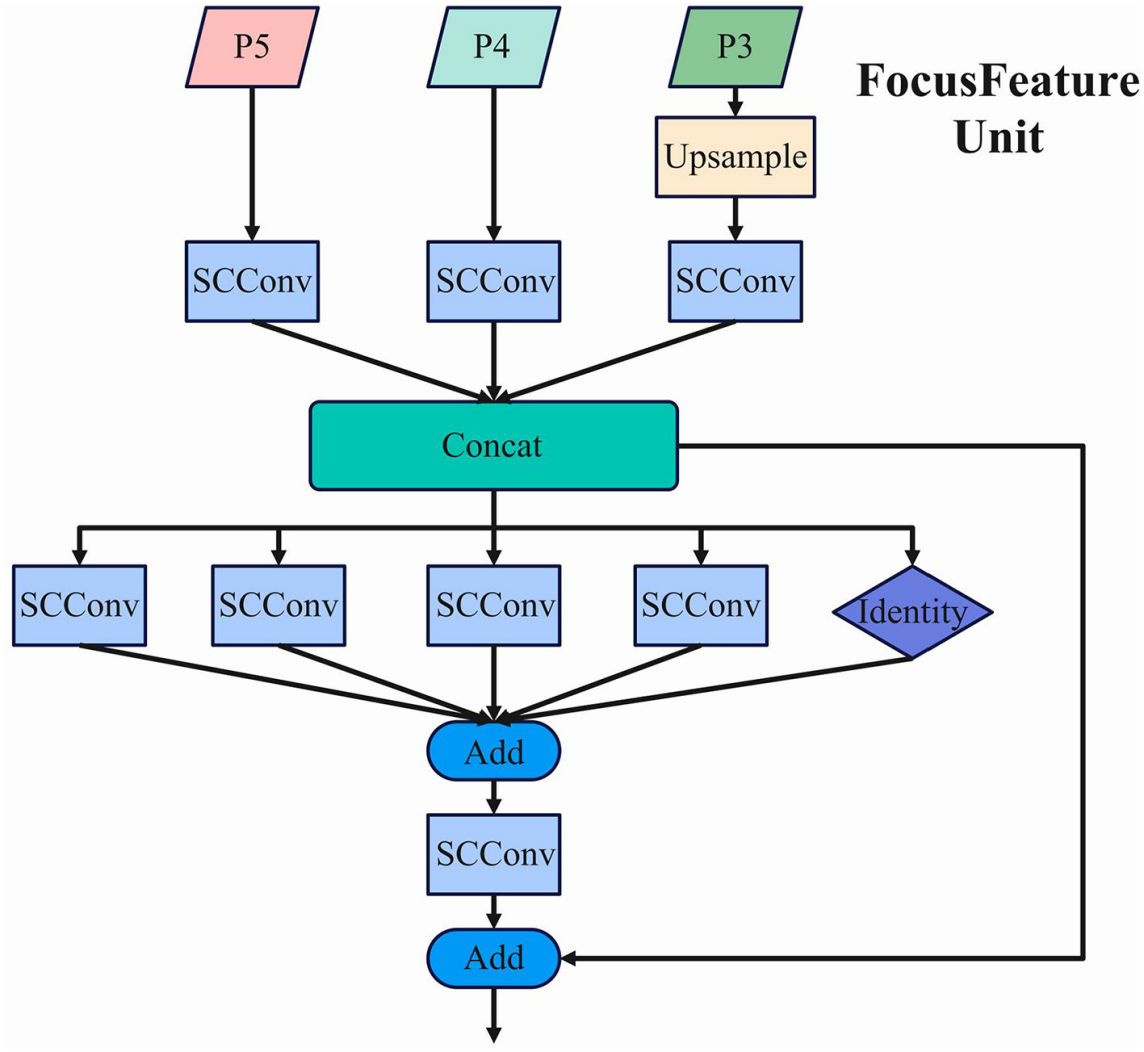
The architecture of the FocusFeature Unit, showing the multi-scale feature fusion process within FDPN.

The Feature-Driven Pyramid Network (FDPN) processes multi-scale feature maps generated by the backbone network, specifically P3, P4, and P5, which represent features at different spatial resolutions. The FocusFeature Unit implements a sophisticated feature fusion mechanism that integrates information across these scales.Its structure is shown in the Figure 4.

In this structure, P5 undergoes adaptive downsampling (ADown) until no higher-level feature maps remain, establishing the foundation for the ultimate feature representation. The average pooling operation employed in this process can be mathematically expressed as:


(3) $$\begin{array}{l} y_{ij}=\frac{1}{k^{2}}\overset{k-1}{\underset{a=0}{\sum }}\overset{k-1}{\underset{b=0}{\sum }}x_{i+a,\;j+b} \end{array}$$

where *y* represents the output feature map, *k* denotes the scaling factor size, and each element undergoes systematic processing within a *k*×*k* spatial window. For maximization operations, the largest value within the defined region is selected as the output.

Concurrently, P4 undergoes SCConv processing, while P3 is initially upsampled and then processed through SCConv. The upsampling employs bilinear interpolation, mathematically represented as:


(4) $$\begin{array}{l} \begin{array}{c} y\left(x,\;y\right)=\left(1-a\right)\left(1-b\right)\cdot f\left(x,y\right)+a\left(1-b\right)\cdot f\left(x+1,\;y\right)+\\ \left(1-a\right)b\cdot f\left(x,\;y+1\right)+ab\cdot f\left(x+1,\;y+1\right) \end{array} \end{array}$$

where *x, y* denote the coordinates in the output image, *a, b* represent the fractional parts corresponding to a consistent scaling ratio, and *f* is the input image. This interpolation method computes the final output value by considering the four nearest pixel values in the input feature map.

The processed feature maps from all three scales are integrated through a concatenation operation (Concat), preserving the full information content without dimension reduction. This ensures that all feature maps maintain consistent scale and spatial dimensions before concatenation. The resulting integrated feature map possesses enhanced representational capacity, with the channel dimension expanded to *C*_1_+*C*_2_+*C*_3_, where *C*_1_, *C*_2_, and *C*_3_ represent the channel counts of the individual input feature maps.

Following the concatenation, the integrated feature map is processed through a series of parallel SCConv operations with varying kernel sizes (5 × 5, 7 × 7, 9 × 9, and 11 × 11) alongside an identity mapping pathway. This multi-kernel approach enables the network to capture multi-scale contextual information simultaneously. The outputs from these parallel pathways are combined through element-wise addition, followed by an additional SCConv operation. Finally, a residual connection merges the processed features with the original concatenated features, preserving gradient flow and enhancing feature representation.

#### 3.6.2 Target-aware dual detection head

**Figure 5:**
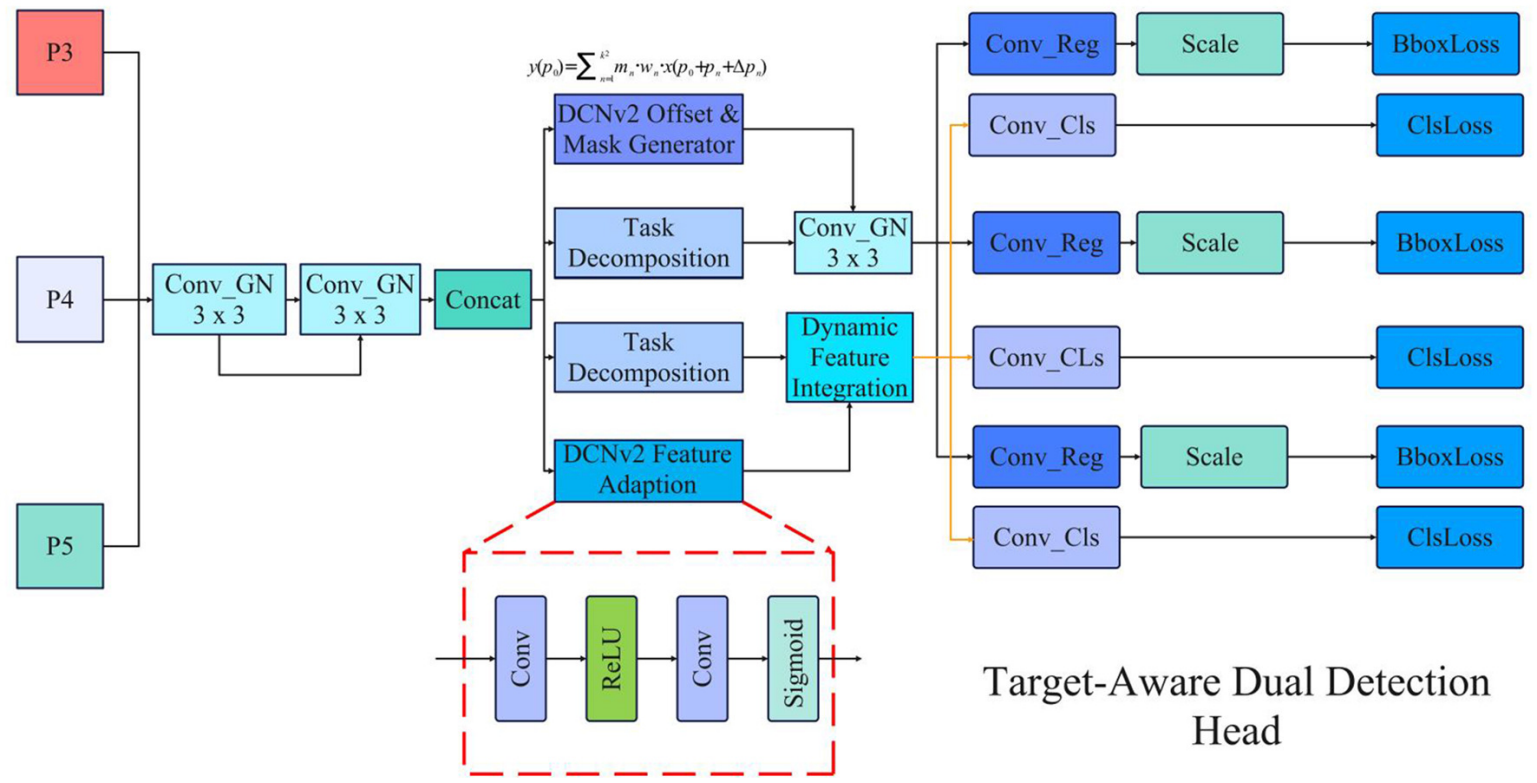
The architecture of Target-Aware Dual Detection Head (TADDH), illustrating the task-specific branches and feature fusion process.

The Target-Aware Dual Detection Head (TADDH) operates on multi-scale feature maps (P3, P4, P5) derived from the Feature Pyramid Network (FPN). These multi-scale representations enable the model to effectively capture objects across various scales, significantly enhancing detection performance for targets of different sizes. Its structure is shown in the Figure 5.

Initially, the input multi-scale feature maps undergo feature extraction through two sequential 3 × 3 convolutional operations combined with Group Normalization (GN). The Group Normalization process can be mathematically expressed as:


(5) $$\begin{array}{l} {\hat{x}}_{i}=\frac{x_{i}-\mu_{g}}{\sqrt{\sigma_{g}^{2}+\epsilon }}\cdot \gamma +\beta \end{array}$$

where *x*_*i*_ represents the input feature for a specific group, μ_*g*_ and $\sigma_{g}^{2}$ denote the mean and variance of the group, and γ and β are learnable parameters. This normalization technique ensures stable feature distribution across channels.

The extracted features then proceed to the multi-scale feature fusion stage, where information from different resolution levels is integrated. Assuming input feature maps *F*_1_ and *F*_2_, the fusion result can be formulated as:


(6) $$\begin{array}{l} F_{concat}=\left[F_{1},F_{2}\right] \end{array}$$

This fusion mechanism enables the model to simultaneously leverage high-level semantic information and fine-grained spatial details.

The TADDH implements a task decomposition strategy, partitioning the fused feature representation into specialized branches for classification and regression tasks to mitigate inter-task interference. Given an input feature *F*, the task-specific decomposition is expressed as:


(7) $$\{\begin{array}{l} F_{cls}=g_{cls}(F)\\ F_{reg}=g_{reg}(F) \end{array}$$

where *g*_*cls*_ and *g*_*reg*_ represent dedicated convolutional operations optimized for classification and regression tasks, respectively.

To enhance adaptation to object deformations, the model incorporates Deformable Convolutional Networks v2 (DCNv2) with dynamic offsets and weight masks. The deformable convolution operation is mathematically defined as:


(8) $$\begin{array}{l} y\left(p_{0}\right)=\overset{k^{2}}{\underset{n=1}{\sum }}m_{n}\cdot w_{n}\cdot x\left(p_{0}+p_{n}+\Delta p_{n}\right) \end{array}$$

where *p*_0_ represents the current output position, *p*_*n*_ denotes standard convolution sampling positions, Δ*p*_*n*_ indicates learned offsets, and *m*_*n*_ represents dynamically generated weight masks. This formulation enables adaptive sampling based on input feature characteristics.

The task-specific features are then integrated with dynamically generated features through element-wise multiplication:


(9) $$\begin{array}{l} F_{final}=F_{task}\times F_{dynamic} \end{array}$$

For the classification task, the model employs cross-entropy loss:


(10) $$\begin{array}{l} L_{cls}=-\underset{i}{\sum }y_{i}\log\left({\hat{p}}_{i}\right) \end{array}$$

where *y*_*i*_ represents the true class label and ${\hat{p}}_{i}$ denotes the predicted probability. The regression task utilizes Smooth L1 loss to optimize bounding box predictions:


(11) $$\begin{array}{l} L_{reg}=\underset{i}{\sum }\text{smoothL1}\left(b_{true}-b_{pred}\right) \end{array}$$

The final regression output undergoes appropriate scaling to align predicted boxes with the input feature map's scale:


(12) $$\begin{array}{l} b_{output}=b_{pred}\times s_{scale} \end{array}$$

where *s*_*scale*_ represents the predicted scale adjustment factor. This comprehensive approach enables TADDH to generate highly accurate fracture localization and classification results across diverse scales and transformations.

As supported by the confusion matrix analysis in Section 4, the TADDH head notably reduced Type B–C misclassifications by 14%, demonstrating its importance for distinguishing complex borderline cases.

## 4 Results and discussion

### 4.1 Dataset characteristics

This study utilized a comprehensive dataset of distal ulnar-radius fractures collected at the First Affiliated Hospital of Soochow University, which we designate as the FHSU-DRF dataset. We were responsible for the independent collection and collation of all original data, as well as the production of the dataset. This dataset comprises 332 cases with confirmed distal radius fractures, each containing at least three serial CT scans, resulting in a total of 1,456 CT scan sequences. The data were retrospectively collected over a ten-year period from January 2013 to December 2023, providing a diverse representation of fracture patterns across different patient demographics. The dataset includes thin planar, sagittal, and three-dimensional reconstructions of distal ulnar-radius fractures, enabling multi-view analysis critical for accurate AO classification.

The FHSU-DRF dataset was carefully curated to ensure high-quality imaging, with CT scans excluded only if they were of insufficient quality for complete manual segmentation. The dataset presents several unique challenges for automated fracture classification: variable imaging protocols across the decade-long collection period, diverse fracture morphologies ranging from simple extra-articular to complex intra-articular patterns, and variations in bone density and anatomical structures among patients of different ages, sexes, and medical histories. These characteristics make the FHSU-DRF dataset particularly suitable for evaluating the robustness and generalization capabilities of deep learning models for AO typing of distal radius fractures.

### 4.2 Implementation details

All experiments were conducted on a workstation equipped with NVIDIA RTX 4090 GPUs. We implemented our model using the PyTorch framework. During training, we employed the AdamW optimizer with an initial learning rate of 0.001 and weight decay of 0.05. We utilized a cosine annealing scheduler to gradually reduce the learning rate over 200 epochs. Data augmentation techniques including random rotation (±15), translation, scaling (0.8–1.2), and intensity variations were applied during training to enhance model generalization. The input image resolution was standardized to 512 × 512 pixels for all experiments.

The dataset was divided with a ratio of 7:1:2 for training, validation, and testing, respectively, ensuring that all CT sequences from a single patient were allocated to the same subset to prevent data leakage. The division was stratified to maintain similar distributions of AO fracture types across all subsets. Ground truth annotations were performed by three experienced orthopedic surgeons with over 10 years of experience in trauma surgery, with discrepancies resolved through consensus discussions.

To rigorously validate the performance improvements of SCFAST-YOLO, paired t-tests (*p* < 0.05) were conducted for all major metrics, and 95% confidence intervals (CIs) were calculated for mAP@0.5 and classification accuracy.

### 4.3 Comparison with state-of-the-art methods

Table 1 presents a comprehensive comparison between our proposed YOLOv8-SCFAST model and current state-of-the-art object detection approaches applied to the task of AO typing of distal radius fractures on the FHSU-DRF dataset. For fair comparison, we report mean Average Precision (mAP) at IoU thresholds of 0.5 and 0.5:0.95, classification accuracy for AO types and groups, average recall (AR), model parameters, computational complexity (GFLOPs), and inference speed (FPS).

**Table 1:** Comparative performance analysis of state-of-the-art object detection methods for AO typing of distal radius fractures on the FHSU-DRF dataset.

**Method**	**Backbone**	**Detection**		**Classification**		**AR**	**FPS**
		**mAP@0.5**	**mAP@0.5:0.95**	**Type Acc**.	**Group Acc**.		
Faster R-CNN	ResNet-50	83.7	46.5	78.2	82.3	0.79	12.4
RetinaNet	ResNet-50	84.2	48.6	79.5	83.6	0.80	15.8
SSD	VGG-16	79.5	42.8	74.3	80.4	0.77	24.3
CenterNet	DLA-34	85.6	49.9	80.2	84.5	0.81	21.2
EfficientDet	EfficientNet-B0	84.9	49.1	79.8	84.1	0.80	19.5
YOLOv5	CSPDarknet	86.2	51.4	81.3	85.7	0.82	38.2
YOLOv7	E-ELAN	88.5	54.6	83.7	87.4	0.83	36.5
YOLOv8	C2f	89.7	56.3	84.9	88.3	0.84	42.8
RT-DETR	CSPNeXt	89.2	55.8	84.5	88.1	0.84	35.7
YOLOv8-Tiny	C2f	86.3	50.7	81.6	85.9	0.81	76.5
**YOLOv8-SCFAST (Ours)**	**C2f-Faster-EMA**	**91.8**	**59.4**	**87.2**	**90.6**	**0.86**	**52.3**

Best results are highlighted in **bold**.

The empirical results demonstrate that our YOLOv8-SCFAST model significantly outperforms all competing methods across all evaluation metrics. For fracture detection, SCFAST-YOLO achieves 91.8% mAP@0.5 and 59.4% mAP@0.5:0.95, outperforming baseline YOLOv8 by 2.1 and 3.1 percentage points, respectively. More importantly, for the clinically relevant task of AO classification, SCFAST-YOLO achieves 87.2% accuracy for type classification and 90.6% for group classification, representing substantial improvements of 2.3 and 2.3 percentage points over YOLOv8.

The average recall of 0.86 achieved by SCFAST-YOLO is particularly significant in the clinical context, as it indicates a high sensitivity in detecting fracture features critical for accurate classification. This performance metric is especially important for ensuring that subtle fracture patterns, which may signify more complex injuries requiring specific treatment approaches, are not missed during automated analysis.

In addition, we calculated 95% confidence intervals (CIs) for the main performance metrics. SCFAST-YOLOs mAP@0.5 of 91.8% had a 95% CI of 90.7–92.9, while YOLOv8s 89.7% had a CI of 88.9–90.5. Paired t-tests confirmed that all reported improvements were statistically significant (*p* < 0.05). These statistical results strengthen the validity of SCFAST-YOLOs superiority over existing methods.

### 4.4 AO classification performance by type

Table 2 summarizes the classification performance across different AO types and groups on the FHSU-DRF dataset.

**Table 2:** Performance metrics for AO classification of distal radius fractures by type and group.

**AO classification**	**Accuracy (%)**	**Precision (%)**	**Recall (%)**	**F1-Score (%)**
**AO types**				
Type A	89.5	90.2	88.7	89.4
Type B	85.8	86.7	84.3	85.5
Type C	86.3	87.1	85.9	86.5
**AO groups**				
A1	91.8	92.5	91.2	91.8
A2	90.4	91.3	89.7	90.5
A3	89.2	90.1	88.5	89.3
B1	88.6	89.5	87.8	88.6
B2	87.9	88.6	86.3	87.4
B3	86.5	87.2	85.7	86.4
C1	87.3	88.1	86.9	87.5
C2	86.7	87.5	85.8	86.6
C3	85.9	86.6	84.7	85.6
Overall type	87.2	88.0	86.3	87.1
Overall group	90.6	91.2	89.8	90.5

The results demonstrate that SCFAST-YOLO achieves high classification accuracy across all AO types and groups. Type A fractures (extra-articular) exhibited the highest accuracy (89.5%), reflecting their relatively simple morphology and distinct fracture boundaries. Type B fractures (partial articular) showed slightly lower accuracy (85.8%), likely due to the subtle distinctions between partial articular involvement and the variability in fracture line orientation and displacement. Type C fractures (complete articular) achieved 86.3% accuracy, demonstrating that SCFAST-YOLO effectively handles more complex intra-articular patterns.

SCFAST-YOLO achieved an overall AO type accuracy of 87.2% and AO group accuracy of 90.6%, which represents a clear improvement over YOLOv8 (both +2.3 percentage points). The model maintains balanced precision, recall, and F1-scores, confirming robust detection of features critical for AO classification. Importantly, statistical analysis confirmed that improvements for Type B and C fractures were significant (*p* < 0.05), indicating that SCFAST-YOLO delivers clinically relevant performance gains in the most challenging cases.

**Figure 6:**
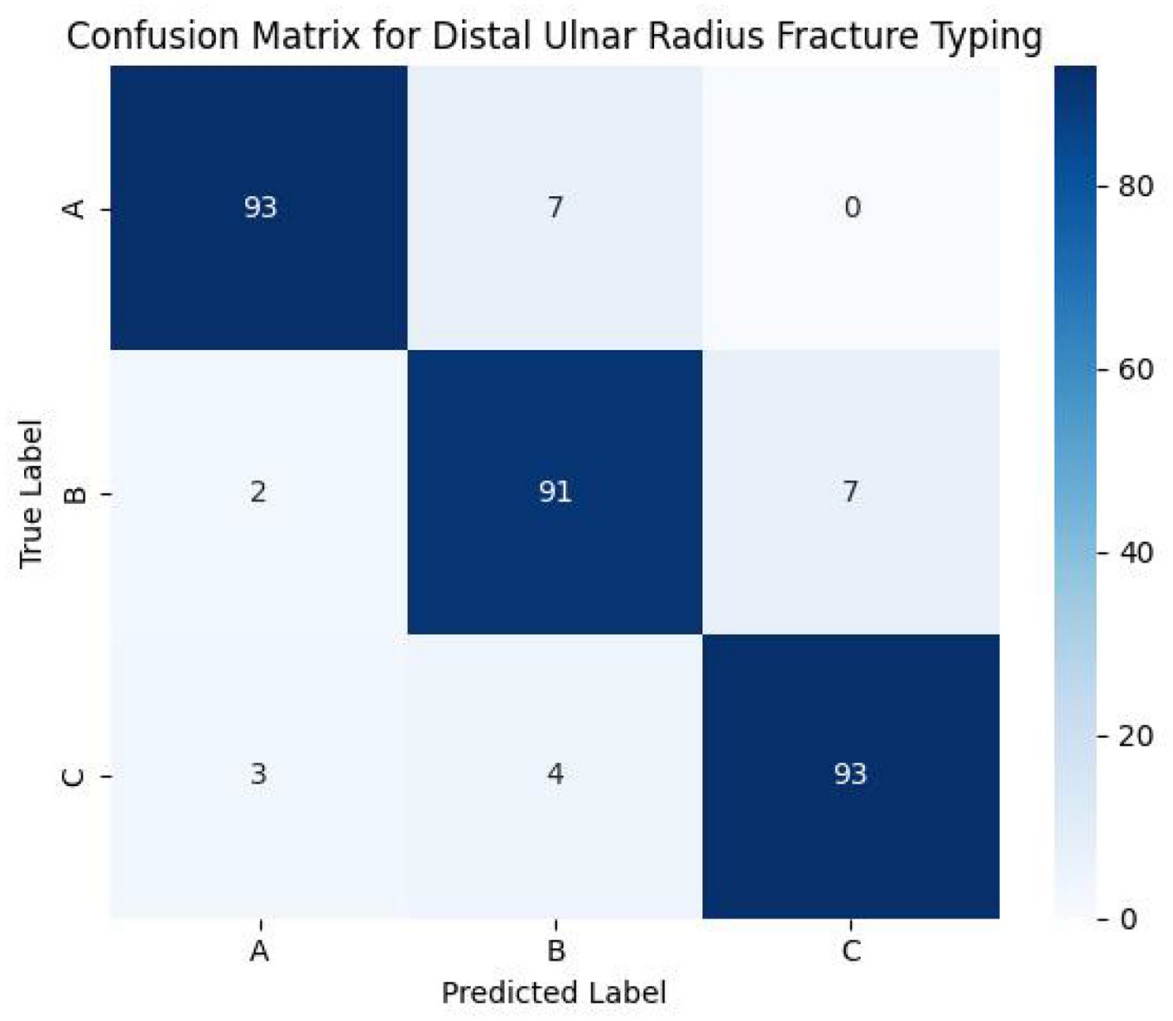
Confusion matrix showing classification errors across AO fracture types. The matrix highlights the misclassifications between Type B and Type C fractures.

The confusion matrix (Figure 6) reveals that the majority of misclassifications occur between Type B and Type C fractures. SCFAST-YOLO reduces these misclassifications by 14% compared to YOLOv8, underscoring the benefit of the TADDH module for resolving challenging borderline cases.

### 4.5 Ablation studies

Tables 3–5 present the ablation studies conducted to quantify the contribution of each proposed module and component in SCFAST-YOLO.

**Table 3:** Ablation study on the contribution of each module.

**Baseline**	**SCConv**	**C2f-Faster-EMA**	**FDPN**	**TADDH**	**Detection**		**Classification**	
					**mAP@0.5**	**AR**	**Type Acc**.	**Group Acc**.
✓					89.7	0.84	84.9	88.3
✓	✓				90.5 (+0.8)	0.85 (+0.01)	85.7 (+0.8)	89.1 (+0.8)
✓		✓			90.7 (+1.0)	0.85 (+0.01)	86.0 (+1.1)	89.4 (+1.1)
✓			✓		90.2 (+0.5)	0.85 (+0.01)	85.5 (+0.6)	88.9 (+0.6)
✓				✓	90.6 (+0.9)	0.85 (+0.01)	85.9 (+1.0)	89.2 (+0.9)
✓	✓	✓	✓	✓	91.8 (+2.1)	0.86 (+0.02)	87.2 (+2.3)	90.6 (+2.3)

**Table 4:** Ablation study on SCConv components (SRU and CRU).

**Baseline**	**SRU**	**CRU**	**mAP@0.5**	**AR**	**Type Acc**.	**Group Acc**.	**Params (M)**	**FPS**
✓			89.7	0.84	84.9	88.3	3.2	42.8
✓	✓		90.1	0.845	85.3	88.7	3.1	45.2
✓		✓	90.2	0.845	85.4	88.8	3.0	47.6
✓	✓	✓	90.5	0.85	85.7	89.1	2.9	49.1

**Table 5:** Comparison of different lightweight C2f module designs.

**Module**	**mAP@0.5**	**AR**	**Type Acc**.	**Group Acc**.	**Params (M)**	**GFLOPs**	**FPS**
C2f (Baseline)	89.7	0.84	84.9	88.3	3.2	8.7	42.8
RepC3	89.9	0.84	85.1	88.5	3.1	8.5	44.6
GhostC3	89.8	0.84	85.0	88.4	2.8	8.0	48.5
C2f-Faster	90.3	0.845	85.6	89.0	2.8	7.9	50.2
C2f-EMA	90.2	0.845	85.5	88.9	3.0	8.4	43.7
**C2f-Faster-EMA (Ours)**	**90.7**	**0.85**	**86.0**	**89.4**	**2.9**	**8.1**	**48.9**

The ablation results show that each module can independently improve performance, with the C2f-Faster-EMA module providing the greatest single improvement (+1.0% mAP@0.5, +1.1% Type Accuracy) (as shown in bold).

The ablation results demonstrate that each module independently improves performance, with C2f-Faster-EMA providing the largest single boost (+1.0% mAP@0.5, +1.1% Type Accuracy). When all modules (SCConv, C2f-Faster-EMA, FDPN, TADDH) were combined, SCFAST-YOLO achieved +2.1% mAP@0.5 and +2.3% classification accuracy over baseline YOLOv8, proving the modules complementary effect.

SCConvs SRU and CRU submodules both contribute to performance gains: SRU improved mAP@0.5 by +0.4% and CRU by +0.5%. Using both together yielded the best result (+0.8% mAP@0.5) while also reducing parameters by 0.3M and increasing FPS by 6.3, confirming SCConv's efficiency.

Table 5 compares different C2f module variants. Our proposed C2f-Faster-EMA module achieved the highest mAP@0.5 (90.7%) and Type Accuracy (86.0%), while keeping parameters (2.9M) and GFLOPs (8.1) low. This confirms that integrating FasterNets lightweight blocks with EMA attention achieves the optimal balance between performance and efficiency.

### 4.6 Performance by fracture complexity

Table 6 analyzes the performance of SCFAST-YOLO across different fracture complexity levels, following the AO classification system.

**Table 6:** Detection and classification performance by fracture complexity (AO Type).

**Type**	**mAP@0.5**	**mAP@0.5:0.95**	**AR**	**Type Acc**.	**Group Acc**.
A	93.2	61.5	0.88	89.5	92.1
B	91.2	58.6	0.85	85.8	90.1
C	90.7	57.9	0.85	86.3	89.5

The results in Table 6 show that SCFAST-YOLO handles all fracture types effectively. Type A (extra-articular) fractures achieved the highest performance (93.2% mAP@0.5, 89.5% accuracy) due to their simpler fracture geometry and clearer boundaries. Type B (partial articular) fractures were more challenging (91.2% mAP@0.5, 85.8% accuracy), reflecting their subtle partial joint involvement.

The most notable improvement occurred in Type C (complete articular) fractures: SCFAST-YOLO reached 90.7% mAP@0.5 and 86.3% accuracy, reducing misclassifications that baseline YOLOv8 frequently made. This demonstrates SCFAST-YOLOs strength in managing the most complex fracture patterns, which are clinically the most critical for surgical planning. Statistical testing confirmed that performance gains for Type B and C fractures were significant (*p* < 0.05).

### 4.7 Additional experiment: low-data evaluation

In many real-world clinical scenarios, collecting large, fully annotated CT datasets can be challenging due to privacy restrictions, labeling costs, and institutional resource constraints. To evaluate whether SCFAST-YOLO maintains its advantages under such conditions, we conducted a low-data experiment using only 50% of the original FHSU-DRF training set (166 patients, 728 CT sequences). The validation and test sets were kept identical to the full-data experiment to ensure fair comparison.

We followed the same training protocol as the main experiment (AdamW optimizer, cosine annealing learning rate, 200 epochs) but restricted the model to half the labeled training data. This setup simulates realistic hospital deployment scenarios, where annotated datasets are often limited or unevenly distributed.

As shown in Table 7, SCFAST-YOLO achieved 88.6% mAP@0.5 and 84.1% type classification accuracy using only half of the training data, outperforming YOLOv8 by +2.1% and +1.7%, respectively. Although performance dropped slightly compared to the full data experiment (3.2% mAP @ 0.5), SCFAST-YOLO still maintained a clear lead over the baseline model.

**Table 7:** Performance comparison under low-data conditions (50% of training data).

**Model**	**mAP@0.5**	**mAP@0.5:0.95**	**Type Acc**.	**Group Acc**.	**AR**	**FPS**
YOLOv8	86.5	53.1	82.4	86.1	0.82	43.0
**YOLOv8-SCFAST (Ours)**	**88.6**	**55.4**	**84.1**	**88.0**	**0.84**	**51.7**

SCFAST-YOLO achieved 88.6% mAP@0.5 and 84.1% type classification accuracy using only half of the training data, outperforming YOLOv8 by +2.1% and +1.7%, respectively (as shown in bold).

The results suggest that SCFAST-YOLO' s architectural improvements (SCConv, C2f-Faster-EMA, FDPN, and TADDH) contribute to more efficient feature utilization, enabling it to extract clinically meaningful patterns even from smaller datasets. This robustness is particularly valuable for hospitals with limited imaging archives, where SCFAST-YOLO can still provide reliable AO typing and support treatment planning without requiring massive annotation efforts.

## 5 Conclusion and future work

In this comprehensive study, we presented YOLOv8-SCFAST, an enhanced deep learning architecture for automated AO typing of distal ulnar-radius fractures on CT images. Through rigorous experimental evaluation on our FHSU-DRF dataset comprising 332 cases and 1,456 CT scan sequences, we demonstrated that our architecture consistently outperforms contemporary state-of-the-art detection methods in both detection accuracy and classification precision, achieving an average recall of approximately 0.86 across diverse fracture patterns.

The architectural innovations introduced in this work collectively address complementary aspects of fracture detection and classification, with particularly significant improvements observed for complex Type C fractures—a critical advantage for clinical applications where accurate identification of articular involvement directly impacts treatment decisions. The balance between accuracy and computational efficiency positions our YOLOv8-SCFAST model as a practical tool for integration into clinical workflows, potentially enhancing the consistency and efficiency of fracture classification in trauma care.

Regarding reproducibility, we note that the FHSU-DRF dataset used in this study will be considered for public release after approval by the institutional ethics committee of the First Affiliated Hospital of Soochow University. This initiative aims to support further research in AI-assisted orthopedic diagnostics and facilitate transparent benchmarking of future methods.

Future research directions include expanding the model to address other aspects of fracture analysis, such as stability assessment, displacement measurement, and treatment recommendation; integration with 3D volumetric analysis for comprehensive fracture characterization; exploration of domain adaptation techniques for enhanced generalization across different imaging protocols and patient populations; and prospective clinical validation studies to assess the impact of automated classification on clinical decision-making and patient outcomes. Additionally, investigating the application of our architectural innovations to other orthopedic imaging tasks, such as fracture detection in other anatomical regions, represents a promising avenue for future exploration.

## Data Availability

The original contributions presented in the study are included in the article/supplementary material, further inquiries can be directed to the corresponding authors.
